# Absence of ultimate controller and investment efficiency: Evidence from China

**DOI:** 10.1371/journal.pone.0287615

**Published:** 2023-06-23

**Authors:** Jidong Qin, Jiawei Liu, Dan Deng

**Affiliations:** 1 Business School, Chengdu University of Technology, Chengdu, China; 2 College of Economics and Management, Nanjing Forestry University, Nanjing, China; Privatuniversität Schloss Seeburg: Privatuniversitat Schloss Seeburg, AUSTRIA

## Abstract

In modern enterprises with a separation of powers, the ultimate controller can effectively influence the implementation of corporate strategy and operational management efficiency, as well as improve corporate governance by monitoring and limiting the management entrenchment effect within enterprises. Based on the information pertaining to ultimate controllers disclosed by enterprises in their annual reports, this study empirically tested whether the absence of the ultimate controller impacts investment efficiency using the data of Chinese A-share listed companies from 2007 to 2020. It was found that the investment efficiency of enterprises without ultimate controllers is relatively lower than those with ultimate controllers. This is reflected in the insufficient investment of enterprises without an ultimate controller. Moreover, the effect is more significant when the financial environment, internal governance environment, and external governance environment of firms are worse. The mechanism analysis demonstrated that the absence of an ultimate controller causes a more severe insider agency problem and a significantly higher degree of financing constraints, which leads to underinvestment and reduces investment efficiency of firms. The economic consequence test also found that the inefficient investment caused by the absence of ultimate controllers would damage the future value of enterprises, but would increase managers’ compensation. Overall, this study suggests that ultimate controllers are an important part of a firm’s internal governance, especially for monitoring management behavior and resolving agency conflicts.

## 1. Introduction

Since Jensen and Meckling(1976) introduced the issue of conflict of interest between shareholders and managers from a principal-agent perspective [1], academic research has generally focused on the first type of agency problem, based on the relationship between shareholders’ and managers’ interests in developed capital markets. At the same time, the inconsistency of interests between controlling and minority shareholders in listed companies in emerging capital markets, such as China, can give rise to opportunistic behavior by controlling shareholders, thus creating a second type of agency problem [2–4]. The pyramid ownership structure and cross-shareholdings exacerbate the separation of control and earnings rights of listed companies and weaken the positive monitoring effect of ultimate controllers on managers in the corporate governance mechanism [5, 6]. Since the implementation of China’s shareholding reform system in 2005, stock trading activity in the capital market has significantly increased, accelerating the change in China’s control market regime. In the era of fragmented shareholdings, the prevalence of shareholder activism and potential hostile takeovers pose a serious threat to the control of ultimate controllers, resulting in a gradual increase in the absence of ultimate controllers and frequent control struggles. The typical cases of control struggles, such as Leishi Lighting, Gome Electrical Appliances, and Vanke, were due to the decentralization of equity that loosened the company’s control, resulting in a conflict of interests between the company’s large shareholders and managers, thus intensifying the competition for the company’s control by internal interest subjects and the risk of hostile acquisition from outside. According to the Wind database, 31 Chinese A-share listed companies lacked an ultimate controller in 2007, accounting for only 3.07% of all the listed companies. As of December 31, 2020, 176 Chinese A-share listed companies did not have an ultimate controller, accounting for 5.64% of all the listed companies.

Recently, an increasing number of listed companies in the Chinese capital market have become the absence of ultimate controllers, attracting the attention of regulatory authorities. At the same time, the issue of how the absence of ultimate controllers affects corporate governance and business development has also been widely discussed in society. However, academic research on this phenomenon is lacking. The existing literature generally focuses on the influence of controlling shareholders’ equity characteristics on managers’ agency motives based on the agency theory [7, 8]. Some scholars argue that the principal-agent relationship between controlling shareholders and managers allows ultimate controllers to exert positive governance effects by strengthening the control of the board of directors, creating effective monitoring over managers, improving corporate incentive mechanisms, and focusing on the long-term value creation of companies [9].

By collecting and collating information on the ultimate controllers of Chinese A-share listed companies, this study examines whether the absence of ultimate controllers leads to insider control struggles due to competition for control from the perspective of the managerial agency problem, resulting in low investment efficiency. Using Chinese A-share listed companies’ data from 2007 to 2020, we found that the absence of ultimate controllers significantly reduces the investment efficiency of enterprises by increasing the agent cost and opportunistic behavior of managers, as well as worsening the degree of corporate financing constraints. This effect is more pronounced when the monitoring effect of internal and external corporate governance mechanisms is worse, and when it is difficult to raise finance.

This study makes several contributions. First, it provides micro-level evidence from China on the role of ultimate controlling shareholders in monitoring and balancing corporate governance [3, 10]. Previous research has concentrated on the specific decision-making and leadership role of ultimate controllers in corporate governance [11, 12]. However, it has not examined the heterogeneous consequences of the absence of ultimate controllers on the investment behavior of managers in a separation of powers situation. This study focuses on the issue of investment efficiency in firms that absence of an ultimate controller, effectively complementing and extending the research on the specific impact of effective controllers on corporate governance and operations processes.

Second, this study interprets the investment decision-making logic of managers from the perspective of internal agency in enterprises with dispersed ownership and the absence of an ultimate controller. Besides, it expands the application of the agency theory in China based on the relationship between the actual controller and the manager. It also provides a solution to the problem of insufficient investment by managers from the perspective of external stakeholders, such as analysts and media, in the context of increasing dispersion in the equity structure of Chinese listed companies.

Third, from a practical perspective, it is instructive for regulators and investors to understand and monitor enterprise investment behavior in the absence of ultimate controllers, and to give full play to the role of internal and external monitoring and governance of enterprises.

## 2. Literature review and hypothesis development

With the gradual separation of management and ownership rights in listed companies, professional managers have entered companies to gain dominant management rights and assume fiduciary responsibility to shareholders by signing a compensation contract with the owners of companies. Their compensation is directly related to the performance of companies. However, managers can use their information advantages regarding the actual operation of enterprises to seek private interests, which makes the moral hazard and adverse selection behavior of managers the classic agency problem that business owners are most concerned about worldwide. In contrast to the relatively decentralized shareholdings of listed companies in Western countries, shareholding concentration in East Asian countries (regions) is relatively high, resulting in effective monitoring and control of managers by interest groups represented by controlling shareholders and ultimate controllers, and improving the efficiency of corporate governance [13–15].

Based on the equity and social capital factor, ultimate controllers can have a substantial impact on the behavior and decision-making of enterprise managers, especially when such controllers directly hold shares, and they are more willing to curb the problem of manager agency from the perspective of long-term operation to enhance the long-term value of enterprises. Therefore, when the shares held by ultimate controllers are gradually diluted, their influence and control over companies’ strategic decisions are weakened, and their ability and motivation to monitor managers’ investment and operations based on long-term operation and value enhancement are gradually diminished. As a result, companies will face a double crisis from both internal and external sources. Internally, the decentralization of companies’ shareholding will result in a lack of strong supervision and control by major shareholders in implementing fiduciary responsibilities and remuneration contracts. Moreover, the cost and punishment cost of moral hazard and adverse selection behavior of managers will be relatively low. Thus, the tendency of managers to take decisions in their self-interest at the cost of shareholders’ interest will be relatively low. Externally, the decentralized shareholding structure of companies in the absence of ultimate controllers is more likely to trigger hostile takeovers by external barbarians, as evidenced by real-life cases that have occurred in recent years, such as the control dispute in Vanke.

Among the corporate governance factors affecting the efficiency of corporate investment, existing studies have focused more on factors, such as shareholding structure, controlling shareholders, managerial characteristics, and internal controls [16, 17], and generally found that good corporate governance helps improve the information environment of firms [18, 19], reduces the financing costs of enterprises [20–22], and improves the investment efficiency of enterprises [23, 24]. Almeida and Wolfenzon (2006) believe that an enterprise’s ultimate controller can use the pyramid equity structure to assist the enterprise in structuring internal capital markets in the face of external credit constraints, integrating internal and external funding needs and alleviating financing constraints [25]. The ultimate controller can also use the pyramid equity structure to effectively reduce the degree of government intervention in state-owned enterprises and improve the efficiency of corporate governance [26, 27].

Therefore, the absence of ultimate controllers reduces the efficiency of corporate governance, increases the role of managers’ power in the daily management of a company, increases the litigation risk faced by the company, and raises the concern of creditors or equity investors about the future business risks of the company [28]. As a result, the company’s financing environment deteriorates and the degree of corporate financing constraints increases. At the same time, the greater the degree of financing constraints, the less self-owned cash flow managers can use at their discretion, resulting in less corporate investment and lower corporate investment efficiency [29].

Overall, there is a risk that the absence of a company’s ultimate controllers will lead to insider control or control struggles, making it difficult for the corporate governance mechanism to function effectively and causing excessive internal friction. Besides, the risk that the absence of ultimate controllers can lead to an increase in the degree of corporate financing constraints will eventually lead to a decrease in corporate investment and in investment efficiency. Based on this analysis, we propose the following research hypothesis:

***Hypothesis 1*: *Ceteris paribus*, *the absence of ultimate controllers will reduce the investment efficiency of an enterprise*.**

## 3. Sample selection and research design

### 3.1 Sample selection

This study examines the impact of the absence of ultimate controllers on firms’ investment efficiency, using Chinese A-share listed companies from 2007 to 2020 as the research subject. The data on the absence of ultimate controllers was manually collected from annual reports of listed companies, and financial data were obtained from the China Stock Market and Accounting Research (CSMAR) database. Following the research practice, we excluded samples of listed financial companies, ST listed companies, and companies with missing relevant data during the sample screening process, resulting in 28,409 firm-year samples. In addition, to eliminate the influence of extreme values, we winsorized the continuous variables at the level of 1%–99%.

Table 1 presents the annual distribution of the sample. It shows that during the sample period from 2007 to 2020, the number of listed companies without an ultimate controller increased from 31 to 176, and the ratio of such companies to the total number of listed companies also increased from 3.07% to 5.64%, indicating an increasing trend annually. The absence of an ultimate controller is increasingly becoming common in this emerging capital market.

**Table 1:** The annual distribution of samples.

Year	Sample	Absence of ultimate controller		Existence of ultimate controller	
		Number	Ratio	Number	Ratio
2007	1009	31	3.07%	978	96.93%
2008	1106	32	2.89%	1074	97.11%
2009	1225	40	3.27%	1185	96.73%
2010	1294	43	3.32%	1251	96.68%
2011	1420	52	3.66%	1368	96.34%
2012	1799	76	4.22%	1723	95.78%
2013	2105	94	4.47%	2011	95.53%
2014	2269	108	4.76%	2161	95.24%
2015	2269	130	5.73%	2139	94.27%
2016	2364	141	5.96%	2223	94.04%
2017	2560	158	6.17%	2402	93.83%
2018	2753	158	5.74%	2595	94.26%
2019	3117	173	5.55%	2944	94.45%
2020	3119	176	5.64%	2943	94.36%
Total	28409	1412	4.97%	26997	95.03%

### 3.2 Variable definition and research design

#### 3.2.1 Absence of an ultimate controller

The China Securities Regulatory Commission requires listed companies to disclose information on their ultimate controller in their annual financial reports. This requirement made it convenient for the study to obtain data. To measure the absence of an ultimate controller, we employed a dummy variable, with the independent variable *AUC*, taking a value of “1” when there is no ultimate controller, and “0” otherwise.

#### 3.2.2 Investment efficiency

The dependent variable is investment efficiency. Based on the Richardson Model [30], we estimated the current investment expenditure with fundamental data lagged for one period and measured investment efficiency using the absolute value of the model residual. A positive residual indicates that the firm has overinvested in the current period; a negative residual indicates that the firm has underinvested. Therefore, the greater the absolute value of the residual, the lower is the investment efficiency of the firm. The model is as follows:

$$\begin{array}{l} {Invest}_{it}=\beta_{0}+\beta_{1}Q_{i,t-1}+\beta_{2}{Lev}_{i,t-1}+\beta_{3}{Cfo}_{i,t-1}+\beta_{4}{Size}_{i,t-1}+\beta_{5}{Ret}_{i,t-1}\\ \;+\beta_{6}{Age}_{i,t-1}+\beta_{7}{Invest}_{i,t-1}+\mu_{t-1}+\gamma_{j}+\varepsilon_{i,t} \end{array}$$ (1)

where *Invest* is the level of investment in the firm, measured as the ratio of cash paid to build fixed, intangible, and other long-term assets to total assets at the beginning of the period. *Q*, *Lev*, *Cfo*, *Size*, *Ret*, and *Age* represent the firm’s Tobin’s Q, gearing, cash flow, size, stock return, and age, respectively. Besides, we added the dummy variables of year fixed effect *μ*_*t*−1_ and industry fixed effect *γ*_*j*_.

#### 3.2.3 Model setting

To test the impact of the absence of an ultimate controller on investment efficiency, we referred to previous studies [31–33], and set the following model for empirical testing:

$$\begin{array}{c} {Inef}_{it}=\beta_{0}+\beta_{1}{AUC}_{i,t}+\beta_{2}{Size}_{i,t}+\beta_{3}{Lev}_{i,t}+\beta_{4}{Cfo}_{i,t}+\beta_{5}{Roa}_{i,t}+\beta_{6}{Growth}_{i,t}\\ +\beta_{7}{PPE}_{i,t}+\beta_{8}{Board}_{i,t}+\beta_{9}{Indir}_{i,t}+\beta_{10}{Dual}_{i,t}+\beta_{11}Top1_{i,t}+\beta_{12}{InsHold}_{i,t}+\beta_{13}{Big4}_{i,t}\\ +\beta_{14}{GDP}_{i,t}+\beta_{15}{Mindex}_{i,t}+\mu_{t}+\gamma_{j}+\varepsilon_{i,t} \end{array}$$ (2)

where *Inef* is the dependent variable of investment efficiency, calculated using Model (1). *AUC* is the independent variable, which is measured as a dummy variable for the absence of an ultimate controller. Considering that the investment efficiency is affected by the internal characteristics and external environment of a firm [34–36], we also controlled these variables: *Size* (measured as the natural logarithm of total assets), *Lev* (measured as the ratio of total liabilities to total assets), *Cfo* (measured as the ratio of free cash flow to total assets), *Roa* (measured as the ratio of net profit to total assets), *Growth* (measured as the growth rate of sales), *PPE* (measured as the ratio of fixed assets to total assets), *Board* (measured as the natural logarithm of the number of directors), *Indir* (measured as the ratio of the number of independent directors to the number of directors), *Dual* (measured as whether the chairperson and CEO are the same person), *Top1* (measured as the shareholding ratio of the largest shareholder), *InsHold* (measured as the shareholding ratio of institutional investors), *Big4* (measured as whether the auditor is one of the four largest international audit firms), *GDP* (measured as the growth rate of the regional gross domestic product), *Mindex* (measured as the index of market development). In addition, we controlled for the year fixed effect, *μ*_*t*_, and industry fixed effect, *γ*_*j*_.

## 4. Empirical analysis

### 4.1 Descriptive statistics

Descriptive statistics for all variables in this study are shown in Table 2. We can find that the dependent variable, *Inef*, has a minimum value of 0 and a maximum value of 0.286, with a standard deviation of 0.0485, indicating a large difference in investment efficiency among different firms. The mean value of the independent variable, *AUC*, is 0.0497, indicating that 4.97% of the listed firms did not have an ultimate controller during the sample period. The statistical results for the remaining variables are consistent with those in the existing literature, with no significant anomalies.

**Table 2:** Descriptive statistics of main variables.

Variable	N	Mean	SD	Min	p50	Max
*Inef*	28409	0.0427	0.0485	0	0.0284	0.2863
*AUC*	28409	0.0497	0.2173	0	0	1
*Size*	28409	22.2063	1.2771	19.7645	22.0404	26.1347
*Lev*	28409	0.4514	0.2035	0.0614	0.4491	0.9027
*Cfo*	28409	0.0476	0.0705	-0.1618	0.0464	0.2480
*Roa*	28409	0.0369	0.0647	-0.2486	0.0351	0.2180
*Growth*	28409	0.1738	0.4480	-0.5916	0.1022	2.9180
*PPE*	28409	0.9525	0.0530	0.6702	0.9663	1.0000
*Board*	28409	2.2570	0.1788	1.7918	2.3026	2.7726
*Indir*	28409	0.3732	0.0531	0.3077	0.3333	0.5714
*Dual*	28409	0.2402	0.4272	0	0	1
*Top1*	28409	0.3430	0.1483	0.0856	0.3214	0.7402
*InsHold*	28409	0.0644	0.0736	0	0.0381	0.3445
*Big4*	28409	0.0602	0.2378	0	0	1
*GDP*	28409	8.1418	3.1032	1.2	7.8	15.8
*Mindex*	28409	7.2905	1.5475	2.92	7.39	10.55

### 4.2 Benchmark regression

The regression results of Model (2) are shown in Table 3 to test the effect of the absence of ultimate controllers on investment efficiency. Column (1) represents the regression outcome without control variables and Column (2) represents the regression outcome with control variables. Table 3 shows that the regression coefficients of the independent variable, *AUC*, in Columns (1) and (2) are 0.0029 and 0.0032, respectively, and both are significant at the 5% level. Given that the mean value of the investment efficiency of sample firms in Table 2 is 0.0427, it is economically significant that the investment efficiency of firms in the absence of ultimate controllers is 6.79%-7.49% lower than the investment efficiency of firms with ultimate controllers. In general, the regression results in Table 3 imply that the absence of ultimate controllers reduces investment efficiency, consistent with our basic hypothesis.

**Table 3:** Benchmark regression.

	(1)	(2)
	*Inef*	*Inef*
*AUC*	0.0029**	0.0032**
	**(2.2503)**	**(2.4921)**
*Size*		-0.0019***
		(-6.1031)
*Lev*		-0.0029
		(-1.5882)
*Cfo*		-0.0071
		(-1.6372)
*Roa*		0.0078
		(1.4426)
*Growth*		0.0254***
		(39.2718)
*PPE*		-0.0513***
		(-9.2777)
*Board*		-0.0077***
		(-4.0305)
*Indir*		-0.0018
		(-0.2920)
*Dual*		0.0042***
		(6.3363)
*Top1*		-0.0004
		(-0.1909)
*InsHold*		0.0216***
		(5.2451)
*Big4*		-0.0021*
		(-1.7012)
*GDP*		-0.0002
		(-0.9959)
*Mindex*		-0.0001
		(-0.6794)
*_cons*	0.0425***	0.1486***
	(146.6175)	(15.7595)
*Year*	Yes	Yes
*Industry*	Yes	Yes
*N*	28409	28409
adj. *R*^2^	0.0345	0.0990

### 4.3 Robustness test

#### 4.3.1 Propensity Score Matching (PSM) method

This study investigated the impact of the absence of an ultimate controller on investment efficiency of firms. The research conclusion could be influenced by endogeneity problems, wherein the decline in investment efficiency may be caused by some unobservable omitted variables rather than the absence of the ultimate controller. To eliminate this potential endogeneity problem, we used all control variables in Model (2) as matching variables and selected matching samples using the nearest neighbor matching (1:1) PSM method. After the first stage of estimating the probability of the absence of the ultimate controller, we obtained 2,764 matched samples. Table 4 shows the differences between the two groups of samples before and after matching, which show that firm size (*Size*), free cash flow (*Cfo*), return on total assets (*Roa*), firm growth capacity (*Growth*), the number of directors (*Board*), proportion of independent directors to the total number of directors (*Indir*), equity concentration (*Top1*), institutional investor shareholding (*InsHold*), auditor services (*Big4*), and the level of economic development of the region in which the firm is located (*GDP*) are significantly different before matching. However after matching, no significant difference between the two sample groups exists.

**Table 4:** Difference test of PSM sample.

Variable	Matched	Mean		t-test	
		Treated	Control	t	p>|t|
*Size*	U	22.10	22.21	-3.160***	0.002
	M	22.09	22.14	-1.120	0.264
*Lev*	U	0.445	0.452	-1.140	0.256
	M	0.444	0.457	-1.610	0.108
*Cfo*	U	0.0370	0.0481	-5.800***	0
	M	0.0374	0.0360	0.520	0.601
*Roa*	U	0.0188	0.0379	-10.83***	0
	M	0.0193	0.0150	1.370	0.172
*Growth*	U	0.143	0.175	-2.670***	0.008
	M	0.142	0.141	0.050	0.958
*PPE*	U	0.953	0.953	0.210	0.833
	M	0.953	0.954	-0.670	0.503
*Board*	U	2.245	2.258	-2.580***	0.01
	M	2.243	2.249	-0.860	0.390
*Indir*	U	0.377	0.373	2.820***	0.005
	M	0.377	0.378	-0.450	0.654
*Dual*	U	0.283	0.238	3.880***	0
	M	0.282	0.257	1.490	0.136
*Top1*	U	0.2252	0.3491	-31.13***	0
	M	0.2263	0.2331	-1.500	0.133
*InsHold*	U	0.0702	0.0641	3.030***	0.002
	M	0.0695	0.0698	-0.0900	0.924
*Big4*	U	0.0786	0.0592	2.990***	0.00300
	M	0.0702	0.0809	-1.070	0.283
*GDP*	U	7.582	8.167	-6.920***	0
	M	7.583	7.538	0.410	0.683
*Mindex*	U	7.301	7.289	0.300	0.767
	M	7.303	7.298	0.0700	0.941

Table 5 presents the regression results of the empirical test using the PSM sample. After using the matched sample, regression coefficients for the independent variable, *AUC*, in Columns (1) and (2) remain positive and statistically significant at the 5% and 10% levels, respectively. This indicates that after eliminating the potential endogeneity problem arising from the omitted variables, the findings of this study are still valid. In other words, our main finding that the absence of an ultimate controller reduces the investment efficiency of firms is robust.

**Table 5:** PSM samples regression.

	(1)	(2)
	Inef	Inef
*AUC*	0.0039**	0.0034*
	**(2.0879)**	**(1.8909)**
*Size*		-0.0006
		(-0.6033)
*Lev*		-0.0104*
		(-1.8906)
*Cfo*		-0.0242*
		(-1.7827)
*Roa*		0.0052
		(0.3768)
*Growth*		0.0221***
		(10.7136)
*PPE*		-0.0185
		(-1.0038)
*Board*		-0.0110*
		(-1.7654)
*Indir*		-0.0376*
		(-1.9559)
*Dual*		0.0082***
		(3.9374)
*Top1*		-0.0252
		(-0.0312)
*InsHold*		-0.0015
		(-0.1199)
*Big4*		-0.0008
		(-0.2011)
*GDP*		-0.0009
		(-1.3456)
*Mindex*		-0.0018**
		(-2.5430)
_cons	0.0406***	0.1307***
	(31.2483)	(4.3980)
*Year*	Yes	Yes
*Industry*	Yes	Yes
*N*	2794	2794
adj. *R*^2^	0.0562	0.1070

#### 4.3.2 Heckman two-stage test

We use the Richardson model to measure investment efficiency, but model errors may cause some observations not to enter the research sample, resulting in the sample self-selection problem. To solve this issue, we adopted the Heckman two-stage method to eliminate the sample self-selection problem. We used the ratio of firms without an ultimate controller in the same industry and the previous year as an exogenous variable for the Heckman test [28, 33]. The regression results are presented in Table 6. Column (1) of Table 6 shows the regression results of the first stage, in which the regression coefficient of the exogenous variable, *Ratio*, is significantly positive, indicating that the absence of an ultimate controller in a firm is affected by the fact that whether other firms in the same industry have an ultimate controller or not. We added the inverse Mills ratio to the regression in the second stage and found that although the regression coefficient of the inverse Mills ratio (*Lambda*) was significantly positive, the regression coefficient of the independent variable, *AUC*, was still positive and significant at the 1% level. This regression result demonstrates that the basic conclusion of this study is valid even after controlling for the sample self-selection problem. As a result, our main finding that the absence of an ultimate controller reduces investment efficiency is robust.

**Table 6:** Heckman two-stage test.

	(1)	(2)
	*AUC*	*Inef*
***Ratio***	5.8980***	
	**(6.2864)**	
***AUC***		**0.0036** ^***^
		**(2.6662)**
*Size*	-0.0307*	-0.0019***
	(-1.8939)	(-5.6270)
*Lev*	-0.0149	-0.0032*
	(-0.1605)	(-1.6474)
*Cfo*	-0.3985*	-0.0096**
	(-1.7236)	(-1.9768)
*Roa*	-0.7543***	-0.0086
	(-3.0269)	(-1.3964)
*Growth*	-0.0533	0.0249***
	(-1.5240)	(34.8086)
*PPE*	-0.0996	-0.0523***
	(-0.3356)	(-8.9878)
*Board*	0.0299	-0.0067***
	(0.2948)	(-3.2852)
*Indir*	0.8399***	0.0069
	(2.6804)	(0.9890)
*Dual*	0.0448	0.0049***
	(1.3305)	(6.7941)
*Top1*	-0.0346***	-0.0004***
	(-25.7647)	(-3.1333)
*InsHold*	0.4724**	0.0279***
	(2.2979)	(6.0410)
*Big4*	0.4419***	0.0023
	(6.9110)	(1.1577)
*GDP*	-0.0329***	-0.0003
	(-2.8798)	(-1.3813)
*Mindex*	-0.0080	-0.0001
	(-0.7247)	(-0.3657)
*Lambda*		0.0111***
		(3.0139)
_cons	-0.6292	0.1319***
	(-1.2384)	(12.9104)
*Year*	Yes	Yes
*Industry*	Yes	Yes
*N*	24461	24461
Pseudo R^2^ /adj. *R*^2^	0.1266	0.1009

#### 4.3.3 Reverse causality problem

The findings of this study may also be influenced by the reverse causality problem, as per which it is not the absence of an ultimate controller that leads to poorer investment efficiency but that firms with poorer investment efficiency are more likely to not have an ultimate controller. To eliminate the impact of this possible reverse causality problem, we regress Model (2) using investment efficiency in a future period instead of investment efficiency in the current period. The regression results are presented in Columns (1) and (2) of Table 7. As shown in Columns (1) and (2) of Table 7, the regression coefficient of the independent variable, *AUC*, remains positive and significant at the 1% level after using future-period investment efficiency as the dependent variable. This indicates that the basic conclusion of this study holds even after considering the possible reverse causality problem. As a result, our main finding that the absence of an ultimate controller reduces investment efficiency is robust.

**Table 7:** Other robust tests.

	Reverse causality problem		Replace dependent variable	
	(1)	(2)	(3)	(4)
***AUC***	0.0046***	0.0041***	0.0036***	0.0036***
	**(3.2280)**	**(2.8479)**	**(2.6341)**	**(2.6243)**
*Size*		-0.0056***		-0.0036***
		(-16.5202)		(-11.0290)
*Lev*		-0.0061***		0.0141***
		(-3.0863)		(7.3375)
*Cfo*		0.0269***		0.0017
		(5.7166)		(0.3603)
*Roa*		-0.0239***		0.0133**
		(-3.7627)		(2.3324)
*Growth*		0.0080***		0.0186***
		(11.5884)		(27.0771)
*PPE*		-0.0200***		-0.0440***
		(-3.3566)		(-7.4945)
*Board*		-0.0023		-0.0075***
		(-1.1155)		(-3.6874)
*Indir*		0.0148**		0.0060
		(2.2598)		(0.9285)
*Dual*		0.0037***		0.0028***
		(5.1635)		(3.9186)
*Top1*		0.0059***		0.0001
		(2.6996)		(0.0687)
*InsHold*		0.0394***		0.0239***
		(8.9875)		(5.4638)
*Big4*		0.0012		-0.0011
		(0.8822)		(-0.8038)
*GDP*		-0.0002		-0.0003
		(-1.0421)		(-1.3526)
*Mindex*		-0.0001		-0.0007***
		(-0.3081)		(-3.3213)
_cons	0.0411***	0.1817***	0.0482***	0.1802***
	(133.7955)	(17.7567)	(158.5897)	(17.9811)
*Year*	Yes	Yes	Yes	Yes
*Industry*	Yes	Yes	Yes	Yes
*N*	24482	24482	28409	28409
adj. *R*^2^	0.0361	0.0649	0.0334	0.0711

#### 4.3.4 Replace the dependent variable

We replace the measurement of the dependent variable, investment efficiency, to test the robustness of the empirical results. Referring to Biddle et al. [37] and Jiang et al. [23], the following model is used to calculate investment efficiency:

$$\begin{array}{c} {Invest}_{it}=\beta_{0}+\beta_{1}Q_{i,t-1}+\mu_{t-1}+\gamma_{j}+\varepsilon_{i,t-1} \end{array}$$ (3)

The dependent variable, *Invest*, and the independent variable, *Q*, are measured in the same way as in Model (1), and we also control for year and industry dummies in the model. Similar to Model (1), we use the absolute value of the residuals from the regression of Model (3) to measure the investment efficiency of firms; the larger the absolute value of the residuals, the less efficient the firm’s investment. After calculating the firm’s investment efficiency according to Model (3), we introduce it into Model (2) for robustness testing. The regression results are shown in Columns (3) and (4) of Table 7. As shown in Columns (3) and (4) of Table 7, the regression coefficient of the independent variable, *AUC*, is still positive and significant at the 1% level. This indicates that after replacing the investment efficiency measure, the findings of this study are still valid. Consequently, our main finding that the absence of an ultimate controller reduces investment efficiency is robust.

## 5. Further analysis

### 5.1 Channel test

In the previous theoretical analysis, we proposed two channels to explain the impact of the absence of an ultimate controller on firms’ investment efficiency. On the one hand, the absence of an ultimate controller limits firms’ ability to access external resources, exacerbates its financial constraints, and limits its ability to invest. On the other hand, the absence of an ultimate controller reduces the monitoring of managers, raises the agency costs of firms, and reduces managers’ willingness to invest. To test whether the above theoretical analysis is valid, we build Models (4) and (5) using financial constraints and agency costs as mediating variables, respectively, to test whether the resource and governance effect channels proposed in the previous theoretical analysis are valid.

$$\begin{array}{c} {Med}_{it}=\beta_{0}+\beta_{1}{AUC}_{i,t}+\beta_{n}{Cotrol}_{i,t}+\mu_{t}+\gamma_{j}+\varepsilon_{i,t} \end{array}$$ (4)

$$\begin{array}{c} {Inef}_{it}=\beta_{0}+\beta_{1}{AUC}_{i,t}+\beta_{2}{Med}_{i,t}+\beta_{n}{Cotrol}_{i,t}+\mu_{t}+\gamma_{j}+\varepsilon_{i,t} \end{array}$$ (5)

where *Med* is the mediation variable. To verify the financing constraints and agency cost effect channels, we use financial constraints (*SA*) and agency costs (*AC*) to measure them, respectively. Specifically, financial constraints are measured using the SA index [38, 39] and agency costs are measured as the ratio of administrative expenses to operating income [40, 41]. *Controls* is the control variable, which is the same as the control variables in the Model (2).

The results of the mediating effect tests are presented in Table 8. Columns (1) and (2) show the results for the financing constraint effect channel. In Column (1), the regression coefficient of the independent variable, *AUC*, is significantly positive, indicating that the absence of an ultimate controller significantly increases a firm’s financial constraints. In Column (2), the regression coefficients for both the financial constraint, *SA*, and the independent variable, *AUC*, are significantly positive, indicating that financial constraints mediate the relationship between the absence of an ultimate controller and investment efficiency of firms. As a result, the regression results in Columns (1) and (2) of Table 8 validate the financing constraint effect channel in the theoretical analysis. In other words, the absence of an ultimate controller exacerbates the financial constraints faced by firms, which, in turn, affects the investment efficiency of firms.

**Table 8:** Channel test.

	(1)	(2)	(3)	(4)
	*SA*	*Inef*	*AC*	*Inef*
***AUC***	0.2425***	**0.0028** ^**^	0.0618***	**0.0028** ^**^
	**(19.1821)**	**(2.1764)**	**(10.4264)**	**(2.1409)**
***SA/AC***		0.0016***		0.0072***
		**(2.6511)**		**(5.6283)**
*Size*	1.0857***	-0.0036***	-0.0259***	-0.0017***
	(358.4988)	(-4.9947)	(-18.2233)	(-5.4655)
*Lev*	-0.0700***	-0.0028	-0.0783***	-0.0023
	(-3.9396)	(-1.5259)	(-9.4102)	(-1.2725)
*Cfo*	-0.0557	-0.0070	-0.1277***	-0.0062
	(-1.2979)	(-1.6169)	(-6.3579)	(-1.4246)
*Roa*	-0.5202***	0.0086	-0.3753***	0.0105*
	(-9.8092)	(1.5945)	(-15.1054)	(1.9405)
*Growth*	0.0163**	0.0254***	-0.0308***	0.0256***
	(2.5497)	(39.2314)	(-10.2912)	(39.5632)
*PPE*	-0.2021***	-0.0509***	-0.1740***	-0.0500***
	(-3.7121)	(-9.2180)	(-6.8221)	(-9.0473)
*Board*	0.0553***	-0.0078***	0.0172*	-0.0079***
	(2.9256)	(-4.0764)	(1.9432)	(-4.0973)
*Indir*	0.3908***	-0.0024	0.0687**	-0.0023
	(6.5396)	(-0.3947)	(2.4544)	(-0.3741)
*Dual*	0.0344***	0.0042***	-0.0011	0.0042***
	(5.2381)	(6.2515)	(-0.3555)	(6.3516)
*Top1*	0.0012***	-0.0006	-0.0003***	-0.0002
	(5.8089)	(-0.2822)	(-3.3296)	(-0.0797)
*InsHold*	0.0405	0.0215***	0.0960***	0.0209***
	(0.9988)	(5.2298)	(5.0544)	(5.0767)
*Big4*	0.1373***	-0.0024*	0.0326***	-0.0024*
	(11.1044)	(-1.8722)	(5.6345)	(-1.8894)
*GDP*	-0.0092***	-0.0002	-0.0032***	-0.0002
	(-4.4877)	(-0.9251)	(-3.3041)	(-0.8859)
*Mindex*	-0.0023	-0.0001	-0.0042***	-0.0001
	(-1.1365)	(-0.6616)	(-4.4102)	(-0.5322)
_cons	-19.8544***	0.1803***	0.8903***	0.1421***
	(-21.1025)	(11.8365)	(20.4573)	(14.9743)
*Year*	Yes	Yes	Yes	Yes
*Industry*	Yes	Yes	Yes	Yes
*N*	28409	28409	28409	28409
adj. *R*^2^	0.9027	0.0992	0.0761	0.1000

Columns (3) and (4) of Table 8 show the results for the agency cost-effect channel. In Column (3), the regression coefficient of the independent variable, *AUC*, is significantly positive, indicating that the absence of an ultimate controller significantly increases agency costs between shareholders and managers. In Column (4), the regression coefficients for both agency costs, *AC*, and the independent variable, *AUC*, are significantly positive, indicating that agency costs play a partially mediating role between the absence of ultimate controllers and investment efficiency. Consequently, the regression results in Columns (3) and (4) of Table 8 validate the agency cost-effect channel in the theoretical analysis. In other words, the absence of ultimate controllers raises agency costs within firms, which, in turn, affects the investment efficiency of firms.

The regression results in Table 8 confirmed the financing constraint and agency cost effect channels proposed in the theoretical analysis. According to the financing constraint effect channel, the absence of an ultimate controller causes firms to face more severe financial constraints, which in turn affects their investment efficiency. This channel implies that the negative impact of the absence of an ultimate controller on the investment efficiency of firms primarily exacerbates underinvestment. To verify this idea, we examine the effect of the absence of ultimate controllers on firms’ underinvestment and overinvestment separately. The regression results are presented in Table 9. The dependent variable in Column (1) is overinvestment and the dependent variable in Column (2) is underinvestment. The regression coefficient of the independent *AUC* in Column (1) is positive but not significant, and the regression coefficient of the independent *AUC* in Column (2) is positive and significant. This implies that the negative impact of the absence of an ultimate controller on investment efficiency is exacerbated primarily by underinvestment, which further confirms the financing constraints effect channel.

**Table 9:** Over-investment or under-investment.

	(1)	(2)
	*OverInv*	*UnderInv*
***AUC***	**0.0039**	0.0033***
	**(1.2285)**	**(3.4860)**
*Size*	-0.0009	-0.0031***
	(-1.2128)	(-13.3760)
*Lev*	0.0002	-0.0093***
	(0.0429)	(-7.0719)
*Cfo*	-0.0001	-0.0127***
	(-0.0084)	(-3.9309)
*Roa*	0.0027	-0.0029
	(0.1861)	(-0.7454)
*Growth*	0.0484***	0.0053***
	(34.1530)	(9.8017)
*PPE*	-0.0891***	0.0078*
	(-7.1019)	(1.7634)
*Board*	-0.0107**	-0.0038***
	(-2.2783)	(-2.6043)
*Indir*	-0.0185	0.0052
	(-1.2532)	(1.1261)
*Dual*	0.0067***	0.0016***
	(4.1337)	(3.2794)
*Top1*	-0.0030	0.0027*
	(-0.5883)	(1.7961)
*InsHold*	0.0310***	-0.0086***
	(3.2646)	(-2.6459)
*Big4*	-0.0069**	0.0009
	(-2.3505)	(0.9207)
*GDP*	-0.0004	-0.0004**
	(-0.8293)	(-2.3863)
*Mindex*	-0.0006	-0.0000
	(-1.1725)	(-0.1333)
_cons	0.1871***	0.1085***
	(8.2635)	(14.9519)
*Year*	Yes	Yes
*Industry*	Yes	Yes
*N*	10454	17955
adj. *R*^2^	0.1626	0.0880

The agency cost effect is another channel through which the absence of an ultimate controller affects investment efficiency. According to the previous theoretical analysis, the absence of an ultimate controller exposes managers to less monitoring, which in turn raises agency costs within firms. At this point, managers may become more aggressive and seek personal gains by building their own empires, or they may become more informed and unwilling to make efforts for their firm. Although the financing constraint effect channel has been shown to be influenced by financial constraints that prevent managers from building personal empires for personal gains, it remains to be seen whether managers become more passive and reduce their work efforts. To verify this idea, we use a firm’s investment opportunity sensitivity to examine the impact of the absence of ultimate controllers on its managers’ efforts [42, 43]. The model is as follows:

$$\begin{array}{c} {Invest}_{it}=\beta_{0}+\beta_{1}Q_{i,t-1}+\beta_{2}{AUC}_{i,t}+\beta_{3}{{AUC}_{i,t}*Q}_{i,t-1}+\beta_{n}{Cotrol}_{i,t-1}+\mu_{t-1}+\gamma_{j}+\varepsilon_{i,t} \end{array}$$ (6)

where *Q* is the Tobin’s Q value of the firm; *AUC* is whether the firm lacks an ultimate controller; and the dependent variable, *Invest*, and control variables, *Control*, are the same as those in Model (1). The regression results are presented in Table 10, which show that the regression coefficient of the multiplication term of AUC*Q is negative and significant at the 10% level. This regression result indicates that the absence of an ultimate controller reduces a firm’s sensitivity to investment opportunities. This suggests that managers make less efforts for work in the absence of an ultimate controller, which further confirms the agency cost-effect channel.

**Table 10:** Investment sensitivity.

	(1)
	Invest
Q	0.0189***
	(38.5642)
AUC	0.0089
	(0.6944)
AUC*Q	-0.0040*
	**(-1.7770)**
Size	0.0024
	(0.9919)
Lev	0.0006
	(0.0372)
Cfo	0.1224***
	(5.6931)
Age	-0.0179***
	(-4.6208)
Ret	0.0028
	(0.6288)
Invest_t-1_	0.0044*
	(1.7000)
_cons	-0.0414
	(-0.7631)
Year	Yes
Industry	Yes
*N*	25145
adj. *R*^2^	0.0650

### 5.2 Heterogeneity test

#### 5.2.1 Heterogeneous influence of the financial environment

The financial environment of a firm can influence the negative impact of the absence of an ultimate controller on its investment efficiency. According to the financing constraint effect channel, one of the major reasons for the decline in investment efficiency is financial constraints caused by the absence of an ultimate controller. Therefore, when the financial environment of a firm is better, its ability to obtain external financial resources is stronger, and the negative impact of the absence of an ultimate controller on investment efficiency is weaker. In contrast, when the financial environment is worse, the negative impact of the absence of an ultimate controller on investment efficiency should be stronger. To verify this idea, we use the financial market development index and the number of local banks to measure the regional financial environment: when the financial market development index is higher and the number of local banks is larger, this implies that the regional financial environment is better [44–46]. Then, we divide the sample into two groups according to their financial environment and regress them. The regression results are presented in Table 11.

**Table 11:** Heterogeneity of the financial environment.

	(1)	(2)	(3)	(4)
	The financial market growth index is higher	The financial market growth index is lower	The number of local banks is large	The number of local banks is small
**AUC**	**-0.0014**	0.0081***	**0.0014**	0.0064***
	**(-0.7676)**	**(4.4248)**	**(0.8383)**	**(3.0475)**
*Size*	-0.0021***	-0.0012***	-0.0022***	-0.0014***
	(-4.7246)	(-2.8102)	(-5.3813)	(-2.8500)
*Lev*	-0.0019	-0.0047*	-0.0055**	0.0003
	(-0.7505)	(-1.8351)	(-2.3003)	(0.0952)
*Cfo*	-0.0130**	-0.0039	-0.0227***	0.0108
	(-2.1457)	(-0.6305)	(-3.8931)	(1.6417)
*Roa*	0.0079	0.0082	0.0105	0.0033
	(1.0504)	(1.0736)	(1.4662)	(0.4031)
*Growth*	0.0253***	0.0255***	0.0255***	0.0253***
	(26.3146)	(29.3154)	(29.5344)	(25.9860)
*PPE*	-0.0701***	-0.0362***	-0.0453***	-0.0583***
	(-8.1211)	(-5.0668)	(-5.9758)	(-7.1662)
*Board*	-0.0038	-0.0106***	-0.0051**	-0.0099***
	(-1.3386)	(-4.0903)	(-1.9715)	(-3.4582)
*Indir*	0.0118	-0.0162*	-0.0028	0.0025
	(1.3357)	(-1.9424)	(-0.3445)	(0.2789)
*Dual*	0.0039***	0.0037***	0.0039***	0.0046***
	(4.3577)	(3.6884)	(4.4332)	(4.5355)
*Top1*	0.0000	-0.0000	-0.0000	0.0000
	(1.1515)	(-1.5083)	(-0.3229)	(0.1561)
*InsHold*	0.0214***	0.0220***	0.0239***	0.0189***
	(3.7181)	(3.7342)	(4.1394)	(3.2156)
*Big4*	-0.0015	-0.0016	-0.0025*	0.0009
	(-0.8125)	(-0.9390)	(-1.6647)	(0.3830)
*GDP*	-0.0005	-0.0004	0.0003	-0.0010***
	(-1.3548)	(-1.4825)	(0.9541)	(-3.0436)
*Mindex*	-0.0001	-0.0016***	0.0002	0.0000
	(-0.2523)	(-5.2146)	(0.4887)	(0.0112)
_cons	0.1608***	0.1417***	0.1383***	0.1521***
	(11.0698)	(11.3448)	(10.7029)	(10.7939)
*Year*	Yes	Yes	*Year*	Yes
*Industry*	Yes	Yes	*Industry*	Yes
*N*	14849	13560	14879	13530
adj. *R*^2^	0.0903	0.1150	0.1141	0.0872

Table 11 shows that when the regional financial market growth index is higher and the number of local banks is large, the regression coefficient of the independent variable, *AUC*, is not significant, and when the regional financial market growth index is lower and the number of local banks is small, the regression coefficient of the independent variable, *AUC*, is significantly positive. This implies that the negative impact of the absence of an ultimate controller on investment efficiency of firms is stronger when firms’ financial environment is worse.

#### 5.2.2 Heterogeneous influence of the governance environment

A firm’s governance environment can also change the negative impact of the absence of an ultimate controller on its investment efficiency. According to the agency cost effect channel, the absence of an ultimate controller reduces the supervision of managers, which, in turn, increases agency costs and ultimately affects investment efficiency. Therefore, when a firm has a better governance environment, managers are better overseen, which reduces the negative impact of the absence of an ultimate controller on investment efficiency. In contrast, when the governance environment is worse, the negative impact is stronger. To test this idea, we measure the internal governance environment of firms using factors, such as whether the chairperson and CEO are the same person [47, 48] and the proportion of independent directors [49, 50]. When the chairperson and CEO are not the same and the proportion of independent directors is higher, the internal governance environment of the enterprise is better. We measure the external governance environment of firms using the number of analysts covering the firms [51, 52] and the number of media outlets reporting on the firms [53, 54]. When the number of analysts covering a firm is high and the number of media outlets reporting on it is high, the external governance environment of the firm is better. Then, we divide the sample into two groups according to the governance environment and regress them. The regression results are presented in Table 12.

**Table 12:** Heterogeneity of the governance environment.

	(1)	(2)	(3)	(4)	(5)	(6)	(7)	(8)
	Chairman and CEO are dual	Chairman and CEO aren’t dual	The proportion of independent directors is high	The proportion of independent directors is low	The number of analysts covered is large	The number of analysts covered is low	The number of media reported is high	The number of media reported is low
**AUC**	**0.0063** ^**^	**0.0021**	**0.0013**	0.0054***	**0.0014**	0.0049***	**0.0027**	**0.0036** ^**^
	**(2.4281)**	**(1.4011)**	**(0.7499)**	**(2.9179)**	**(0.7275)**	**(3.0137)**	**(1.3702)**	**(2.1120)**
*Size*	-0.0015**	-0.0020***	-0.0026***	-0.0010**	-0.0015***	-0.0035***	-0.0022***	-0.0020***
	(-2.0912)	(-5.8445)	(-6.1245)	(-2.2715)	(-3.0982)	(-7.9357)	(-4.6556)	(-4.7066)
*Lev*	0.0016	-0.0045**	0.0001	-0.0056**	-0.0002	-0.0039*	-0.0036	-0.0028
	(0.4139)	(-2.2005)	(0.0202)	(-2.2244)	(-0.0593)	(-1.8330)	(-1.2792)	(-1.2177)
*Cfo*	-0.0042	-0.0077	-0.0098	-0.0031	0.0134**	-0.0198***	-0.0002	-0.0149**
	(-0.4399)	(-1.5811)	(-1.5896)	(-0.5071)	(1.9867)	(-3.5474)	(-0.0382)	(-2.5181)
*Roa*	0.0055	0.0064	0.0151**	0.0003	-0.0241**	0.0272***	-0.0022	0.0121*
	(0.5025)	(1.0289)	(2.0240)	(0.0390)	(-2.5666)	(4.2178)	(-0.2570)	(1.7752)
*Growth*	0.0296***	0.0241***	0.0262***	0.0247***	0.0343***	0.0172***	0.0271***	0.0238***
	(20.6919)	(33.2814)	(28.6182)	(26.8749)	(33.8839)	(21.2777)	(27.9518)	(27.4188)
*PPE*	-0.0639***	-0.0498***	-0.0473***	-0.0558***	-0.0668***	-0.0338***	-0.0655***	-0.0385***
	(-4.4811)	(-8.3590)	(-6.0436)	(-7.1286)	(-8.0650)	(-4.6900)	(-7.7630)	(-5.2437)
*Board*	-0.0015	-0.0093***	-0.0106***	0.0003	-0.0106***	-0.0055**	-0.0093***	-0.0055**
	(-0.3285)	(-4.4518)	(-4.5270)	(0.0868)	(-3.7956)	(-2.0989)	(-3.2999)	(-2.0530)
*Indir*	-0.0059	0.0016	-0.0096	0.0930	-0.0063	0.0047	-0.0113	0.0079
	(-0.4437)	(0.2274)	(-1.0302)	(0.9129)	(-0.7298)	(0.5710)	(-1.2661)	(0.9367)
*Dual*			0.0036***	0.0047***	0.0066***	0.0010	0.0055***	0.0030***
			(3.8834)	(4.8980)	(6.6889)	(1.1565)	(5.1251)	(3.4930)
*Top1*	0.0070	-0.0020	0.0021	-0.0035	-0.0046	0.0033	0.0009	-0.0003
	(1.5195)	(-0.8649)	(0.7366)	(-1.2297)	(-1.5215)	(1.2152)	(0.2933)	(-0.1220)
*InsHold*	0.0230**	0.0213***	0.0249***	0.0200***	0.0087	0.0368***	0.0248***	0.0155**
	(2.4837)	(4.6509)	(4.1458)	(3.5355)	(1.5603)	(4.6241)	(4.3074)	(2.5055)
*Big4*	-0.0034	-0.0018	-0.0027	-0.0014	-0.0031**	-0.0007	-0.0020	-0.0029
	(-0.9858)	(-1.3850)	(-1.5369)	(-0.8076)	(-1.9960)	(-0.3010)	(-1.1357)	(-1.5192)
*GDP*	-0.0003	-0.0002	-0.0008**	0.0003	-0.0002	-0.0003	-0.0009***	0.0005*
	(-0.4914)	(-0.9040)	(-2.5100)	(1.0397)	(-0.4885)	(-1.0414)	(-2.8960)	(1.6931)
*Mindex*	0.0002	-0.0002	0.0000	-0.0004	-0.0003	0.0000	-0.0006*	0.0003
	(0.3072)	(-0.9578)	(0.1159)	(-1.2359)	(-1.0638)	(0.0599)	(-1.9517)	(0.9614)
_cons	0.1355***	0.1549***	0.1717***	0.0835**	0.1650***	0.1571***	0.1873***	0.1219***
	(5.9417)	(15.0215)	(13.1199)	(2.2478)	(11.5188)	(11.8438)	(13.3787)	(9.1189)
*Year*	Yes	Yes	Yes	Yes	Yes	Yes	Yes	Yes
*Industry*	Yes	Yes	Yes	Yes	Yes	Yes	Yes	Yes
*N*	6825	21584	13925	14484	15033	13376	13695	14360
adj. *R*^2^	0.1040	0.0960	0.1082	0.0929	0.1176	0.0825	0.0988	0.0972

Columns (1) to (4) of Table 12 show the differential impact of the absence of an ultimate controller on investment efficiency of firms in different internal governance environments. As can be seen, when the chairperson and CEO are not the same person and the proportion of independent directors is relatively high, the regression coefficient of the independent variable, *AUC*, is not significant. However, when the chairperson and CEO are the same person and the proportion of independent directors is relatively lower, the regression coefficient of the independent variable, *AUC*, is significantly positive. This finding indicates that the negative effect of the absence of an ultimate controller on investment efficiency is stronger when a firm’s internal governance environment is worse.

Columns (5) to (8) of Table 12 show the differential impact of the absence of an ultimate controller on investment efficiency in different external governance environments. As can be seen, the regression coefficient of the independent variable, *AUC*, is not significant when the number of analysts covering a firm and number of media outlets reporting on it is relatively high. However, when the number of analysts covering a firm and number of media outlets reporting on it is relatively low, the regression coefficient of the independent variable, *AUC*, is significantly positive. This indicates that the negative effect of the absence of an ultimate controller on investment efficiency is stronger when a firm’s external governance environment is worse.

### 5.3 Economic consequence

Our main findings suggest that the absence of ultimate controllers raises the financial constraints and agency costs of firms, which decreases managers’ ability and willingness to make investments and eventually decreases investment efficiency. To verify whether managers and firms suffer losses due to the decline in investment efficiency, we use future compensation (*Salary*) and Tobin’s Q value (*Q*) to investigate the economic consequences of the negative impact of the absence of an ultimate controller on firms’ investment efficiency.


$${Salary/Q}_{it+1}=\beta_{0}+\beta_{1}{Inef}_{i,t}+\beta_{2}{AUC}_{i,t}+\beta_{3}{{AUC}_{i,t}*Inef}_{i,t}+\beta_{n}{Cotrol}_{i,t}+\mu_{t}+\gamma_{j}+\varepsilon_{i,t}$$ (7)

Table 13 presents the economic consequences of the absence of ultimate controllers on investment efficiency. Specifically, the dependent variable in Column (1) is managers’ compensation in the forward period. The regression results show that the regression coefficient of the interaction term of the absence of an ultimate controller and investment efficiency (AUC*Inef) is significantly positive. This indicates that although the absence of an ultimate controller reduces investment efficiency, managers’ compensation still increases. The dependent variable in Column (2) is Tobin’s Q in the forward period. The regression results show that the regression coefficient of the interaction term of the absence of an ultimate controller and investment efficiency (AUC*Inef) is significantly negative. This indicates that the absence of an ultimate controller not only reduces investment efficiency but also reduces the market value of firms. Overall, the results in Table 13 suggest that the negative effect of the absence of an ultimate controller on investment efficiency reduces the market value of firms, but increases managers’ compensation packages.

**Table 13:** Economic consequence.

	(1)	(2)
	*Salary*_*t+1*_	*Q*_*t+1*_
*Inef*	-0.1100	0.0589
	(-1.3085)	(0.4830)
*AUC*	0.1187***	-0.0164
	(3.3024)	(-0.3151)
AUC*Inef	0.6210*	-0.8513*
	**(1.8321)**	**(-1.7134)**
*Size*	0.3962***	-0.5491***
	(49.5801)	(-47.5759)
*Lev*	-0.3029***	0.3775***
	(-7.6716)	(6.5847)
*Cfo*	0.3802***	0.8565***
	(6.0459)	(9.4349)
*Roa*	1.0372***	0.7272***
	(11.5768)	(5.6232)
*Growth*	0.0203**	-0.0834***
	(2.3039)	(-6.5289)
*PPE*	0.1980	-0.6684***
	(1.5862)	(-3.7074)
*Board*	0.1632***	-0.0558
	(3.6515)	(-0.8683)
*Indir*	0.0402	0.3232*
	(0.3292)	(1.8407)
*Dual*	0.0483***	-0.0023
	(3.6465)	(-0.1192)
*Top1*	-0.2900***	0.4672***
	(-4.8617)	(5.4239)
*InsHold*	0.3192***	2.0417***
	(4.7775)	(21.1179)
*Big4*	0.1811***	-0.0106
	(5.0227)	(-0.2061)
*GDP*	-0.0482***	-0.0904***
	(-23.5170)	(-30.6866)
*Mindex*	-0.0226***	0.1520***
	(-4.8191)	(22.4201)
*_cons*	6.1882***	13.9952***
	(24.3470)	(38.1286)
*Year*	Yes	Yes
*Industry*	Yes	Yes
*N*	24018	23554
adj. *R*^2^	0.6743	0.5368

## 6. Conclusions

Ultimate controllers are an important part of corporate governance in modern enterprises with separation of powers. However, the absence of ultimate controllers in listed companies has become increasingly common in the Chinese capital markets. Few studies have investigated how this phenomenon affects managerial behavior and corporate value. This study investigates the impact of the absence of ultimate controllers on firms’ investment efficiency.

Using a sample of Chinese A-share listed companies from 2007 to 2020, this study examines the impact of the absence of ultimate controllers on investment efficiency. We found that firms without an ultimate controller have relatively lower investment efficiency than firms with an ultimate controller. This is mainly reflected in the underinvestment of firms without an ultimate controller. This effect is also economically significant, with firms without an ultimate controller being 6.79%-7.49% less efficient in terms of investment than firms with an ultimate controller. The study also found that this effect is stronger when the financial environment, internal governance environment, and external governance environment of firms are worse.

Further research finds that the absence of an ultimate controller causes a more serious insider agency problem and a significant increase in corporate financing constraints, which leads to underinvestment and lowers investment efficiency. Further, the economic consequence test found that the inefficient investment brought about by the absence of ultimate controllers will damage the future value of enterprises but will increase managers’ compensation.

Overall, this study suggests that ultimate controllers are an important part of the effective functioning of corporate governance in enterprises, as well as a means of monitoring management behavior and reducing agency costs. To prevent issues like insider control from happening, listed companies should address the issue of the absence of an ultimate controller and actively monitor their managers’ behavior. Regulators should actively pay attention to the issue of ultimate controllers of listed companies, supervise the behavior of listed companies from the perspective of institutional arrangements, protect investors’ interests, and act as a good gatekeeper of the capital market.

Meanwhile, this study still has some limitations. First, it treats the issue of companies lacking an ultimate controller as homogeneous, however, companies may not have an ultimate controller for many reasons, and the corporate governance of companies may be different for different reasons. Future research could begin from this perspective and discuss different economic results. Second, the research sample of this study was limited to a single institutional environment of China. Future research should consider cross-country samples and examine corporate governance issues in the absence of ultimate controllers in different economic systems.
