# Correction: Absence of ultimate controller and investment efficiency: Evidence from China

**DOI:** 10.1371/journal.pone.0342026

**Published:** 2026-01-30

**Authors:** Jidong Qin, Jiawei Liu, Dan Deng

The corresponding author’s email address is incorrect. Jiawei Liu’s email address is: 15802847066@163.com.
